# Identifying Patients With Coronary Artery Disease Using Rest and Exercise Seismocardiography

**DOI:** 10.3389/fphys.2019.01211

**Published:** 2019-09-24

**Authors:** Parastoo Dehkordi, Erwin P. Bauer, Kouhyar Tavakolian, Vahid Zakeri, Andrew P. Blaber, Farzad Khosrow-Khavar

**Affiliations:** ^1^Electrical and Computer Engineering Department, Biomedical Department, The University of British Columbia, Vancouver, BC, Canada; ^2^Heart Force Medical Inc., Vancouver, BC, Canada; ^3^School of Electrical Engineering and Computer Science, University of North Dakota, Grand Forks, ND, United States; ^4^Biomedical Physiology and Kinesiology Department, Simon Fraser University, Vancouver, BC, Canada

**Keywords:** coronary artery disease, seismocardiography (SCG), electrocardiograph (ECG), exercise stress test, heart mechanical activity

## Abstract

Coronary artery disease (CAD) is the most common cause of death globally. Patients with suspected CAD are usually assessed by exercise electrocardiography (ECG). Subsequent tests, such as coronary angiography and coronary computed tomography angiography (CCTA) are performed to localize the stenosis and to estimate the degree of blockage. The present study describes a non-invasive methodology to identify patients with CAD based on the analysis of both rest and exercise seismocardiography (SCG). SCG is a non-invasive technology for capturing the acceleration of the chest induced by myocardial motion and vibrations. SCG signals were recorded from 185 individuals at rest and immediately after exercise. Two models were developed using the characterization of the rest and exercise SCG signals to identify individuals with CAD. The models were validated against related results from angiography. For the rest model, accuracy was 74%, and sensitivity and specificity were estimated as 75 and 72%, respectively. For the exercise model accuracy, sensitivity, and specificity were 81, 82, and 84%, respectively. The rest and exercise models presented a bootstrap-corrected area under the curve of 0.77 and 0.91, respectively. The discrimination slope was estimated 0.32 for rest model and 0.47 for the exercise model. The difference between the discrimination slopes of these two models was 0.15 (95% CI: 0.10 to 0.23, *p* < 0.0001). Both rest and exercise models are able to detect CAD with comparable accuracy, sensitivity, and specificity. Performance of SCG is better compared to stress-ECG and it is identical to stress-echocardiography and CCTA. SCG examination is fast, inexpensive, and may even be carried out by laypersons.

## 1. Introduction

Coronary artery disease (CAD) is the most common cause of death worldwide (GBD 2017 Disease and Injury Incidence and Prevalence Collaborators, 2018). Risk factors for CAD are high LDL cholesterol, low HDL cholesterol, high blood pressure, family history, diabetes, smoking, age, and obesity. These risk factors may cause atherosclerotic plaques within the coronary arteries. When the plaques build up they may narrow or even occlude the vessel. As a result, the oxygen supply to the heart muscle is reduced and symptoms like angina pectoris, shortness of breath or fatigue may occur. Severe complications of CAD are myocardial infarction, ventricular fibrillation, heart failure, and death.

Once symptoms of CAD occur, the affected patients may consult a cardiologist, who will perform a rest and exercise electrocardiography (ECG). In case the ECG is pathologic, further tests, such as coronary angiography and coronary computed tomography angiography (CCTA) are performed in order to figure out the location, extent, and degree of coronary occlusion (Ashley and Niebauer, 2004).

Rest and stress ECG are extensively used for diagnosis of CAD. However, in many cases diagnosis of CAD using the morphology of a rest ECG is not straightforward, especially when the disease is in an early stage. Compared to the rest ECG, the stress ECG shows better sensitivity regarding CAD diagnosis, although sensitivity is still below 70% (Al-Shehri et al., 2011).

Cardiac imaging techniques (coronary angiography, CCTA) are currently the gold standard for the diagnosis of CAD. However, these techniques are expensive, only available in hospitals and in the case of coronary angiography, invasive (Al-Shehri et al., 2011).

Many patients with CAD are asymptomatic. It would explain why this disease is the leading cause of death globally. To lower the overall mortality rate of CAD, a technology is required, which is inexpensive, easy to use, fast, reliable and accurate. As a result, a great interest in alternative techniques has been promoted to simplify the procedure of detecting CAD (for example recently a method has been suggested for detecting CAD using the sound of turbulent blood flow induced by coronary stenosis Schmidt et al., 2015; Winther et al., 2016, 2018; Thomas et al., 2017). Among them a technology using seismocardiography (SCG) for detecting CAD has the potential to fulfill these requirements.

SCG is a non-invasive technology for capturing the acceleration of the chest induced by the contraction and relaxation of the myocardium. The acceleration is recorded dorsoventrally, back to front, using an accelerometer placed on the sternum, close to the xiphoid. SCG was initially recommended, in the early 1960s, for monitoring heart rate variability (Baevskii et al., 1964). In the late 80s and early 90s, SCG was used as a technology for measuring the myocardium motion during ventricular contraction and during early and late ventricular filling (Salerno and Zanetti, 1990; Zanetti and Salerno, 1991). A study conducted by Crow et al. later, confirmed that the fiducial points of the dorsoventral SCG were associated with aortic and mitral valve opening and closure events (Zanetti et al., 1991; Crow et al., 1994).

Considering the well-studied relation between heart wall motion and acute or chronic ischemia caused by CAD (Tennant and Wiggers, 1935; Chen et al., 1980; Morganroth et al., 1980), Salerno et al. suggested SCG as a non-invasive technology for detecting coronary artery disease (Salerno et al., 1990; Salerno and Zanetti, 1991). To investigate the seismographic changes associated with coronary artery stenosis and ischemia caused by decreased coronary blood flow, in a study conducted by Salerno et al. 35 patients were studied during coronary angioplasty (Salerno et al., 1991). The findings were consistent with the hypothesis that the SCG changes were due to ischemic changes in ventricular wall motion.

Several studies were later conducted to assess the ability of exercise SCG for detecting CAD. Salerno et al. studied the morphology of exercise SCG in patients with ≥50% coronary artery stenosis (Salerno and Zanetti, 1990). Changes in the morphology of SCG prior to and immediately after exercise were reported as being significant during isovolumetric contraction up to the occurrence of aortic valve opening. Their findings suggested that exercise SCG in conjunction with 12-channel ECG improved the sensitivity of detection of coronary artery stenosis compared to ECG alone.

In this study, we proposed a new automatic and non-invasive methodology to identify patients with CAD based on the analysis of both rest and exercise SCG. The SCG signals were recorded before and immediately after exercise using an accelerometer mounted on the chests in the supine position. Patients with more than 50% occlusion in at least one of their coronary arteries, diagnosed by coronary angiography, were considered as part of the CAD group. This method could offer new possibilities for monitoring CAD with a very simple procedure outside the clinical setting.

## 2. Materials and Methods

### 2.1. Data Set

The current manuscript presents a new methodology for detecting coronary artery disease using the data set collected in 1988–92. The protocol was approved by the human subject research committee of Abbott North-western Hospital, Minneapolis, MN, on March 11, 1988. The study was carried out in accordance with the recommendations of the research committee of Abbott North-western Hospital, Minneapolis, MN. All subjects gave written informed consent in accordance with the Declaration of Helsinki.

Two hundred and four participants were enrolled in the study. All participants underwent a treadmill exercise following the Bruce protocol. SCG and 12-lead ECG signals were recorded in the supine position just prior to exercise (rest recording), immediately after returning to the supine position at the end of exercise (post-exercise recording) and again at the end of the recovery period (recovery recording). Standing 12-lead ECG signals were obtained during the exercise as well.

The SCG signals were recorded using an ultra low-frequency piezoelectric crystal accelerometer (Seismed Instruments, Inc., Minneapolis, Minn) with a linear response between 0.3 and 800 Hz and a sensitivity of 1.0 V/g. The accelerometer was placed on the sternum close to the xiphoid process. Both SCG and ECG signals were sampled at 250 Hz.

Participants were classified as having coronary artery disease if ≥50% stenosis was present in at least one coronary artery. A subgroup of participants had a coronary angiogram. For patients without an angiogram, the probability of coronary artery disease was estimated using the Framingham prospective risk score (D'Agostino et al., 2008). Participants with ≤2% probability of coronary disease were assumed to have <50% stenosis in all coronary arteries.

Among the 204 participants enrolled for study, 19 participants (9.3%) were excluded from the study due to lack of information from angiography, very poor quality ECG or SCG signals, or unwillingness to continue the study. Of the remaining 185 participants, 148 patients (80.0%) underwent the coronary angiography. Significant CAD was found in 117 out of 148 (79.1%) patients while in 31 out of 148 (20.9%) patients no significant CAD reported by coronary angiography.

The subgroup of participants without an angiogram, 37 out of 185 (20.0%), had an estimated CAD risk ≤2%. Among them, 10 out of 37 were sent for Thallium stress test and all had a negative result; 27 out of 37 were sent for ECG stress test and 22 out of 27 had a negative result. Five patients with positive ECG stress test were excluded from the study. It resulted in a total of 180 participants included in the further analysis.

When including the 32 out of 180 (20.0%) individuals with an estimated risk of CAD ≤2% into the calculation, a total of 65 (35.7%) had no significant CAD and 117 (64.2%) had significant CAD with an occlusion rate ≥50% in at least one coronary artery. The average age of the participants was 55 ± 11 years: 59 ± 9 years for the patients with CAD and 48 ± 11 years for the participants with no significant CAD. There were 129 males and 56 females among participants.

More information and details about the data set can be found elsewhere (Salerno et al., 1992).

### 2.2. Data Processing

**Figure 1:**
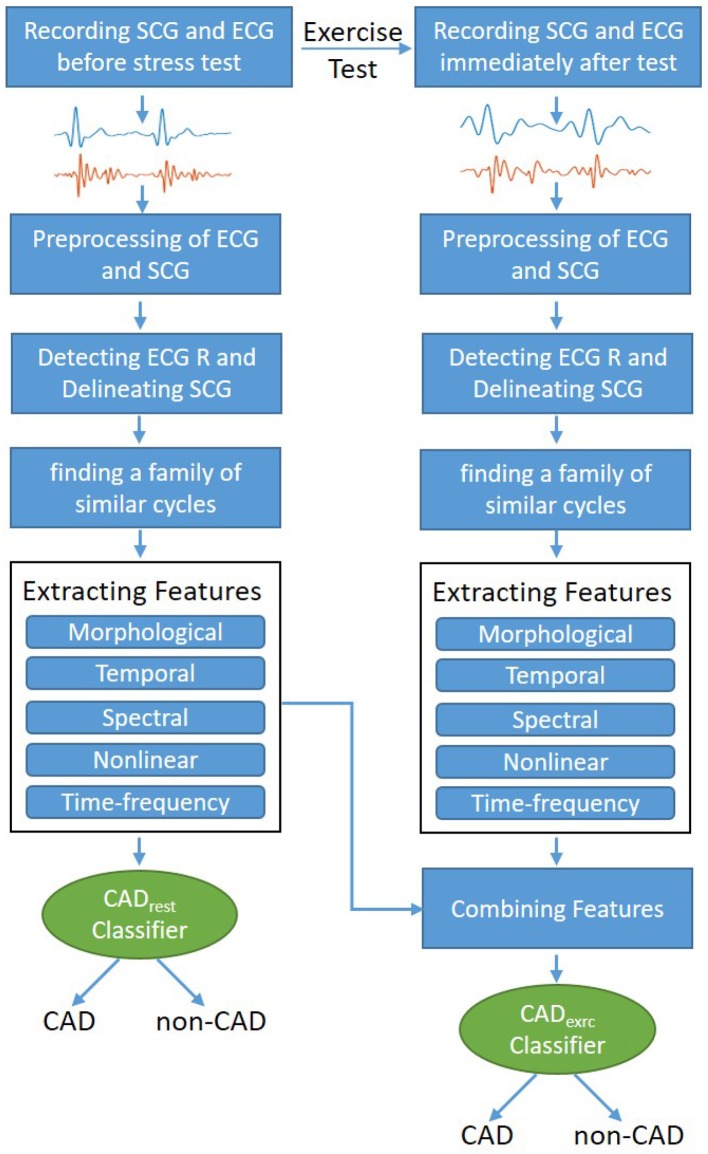
Different steps of methodology designed and developed to identify patients with CAD from control group.

The methodology suggested and developed in this study was depicted in Figure 1. After preprocessing, an algorithm was independently applied to the rest and exercise SCG recordings to form families of cycles with similar morphology. Several features were then extracted from each family. Later, two binary multivariate logistic regression classifiers were developed: the rest CAD classifier (CAD_*rest*_) was trained based on features extracted from rest SCG cycles and the exercise CAD classifier (CAD_*exrc*_) was developed over a data set that included features extracted from both rest and exercise SCG cycles. Leave-one-subject-out method was employed to validate the classifiers.

#### 2.2.1. Preprocessing

To remove baseline wandering, a zero-phase high-pass Butterworth filter with an order of 5 and the cut-off frequency of 0.5 Hz was applied to the rest and exercise SCG signals. Subsequently, the average of SCG signals was removed and the zero-mean signals were normalized between –1 and 1.

#### 2.2.2. Algorithm for Forming the Family of Cycles

Within a subject, the morphology and also the frequency components of the SCG recording varied from one cycle to another. This variation could be mainly due to the effects of breathing (Tavakolian et al., 2008). Accordingly, instead of analyzing all cycles of each SCG signal, only very similar cycles, grouped into families, were included in the further analysis.

The procedure of finding the family of similar cycles involved the following steps:

- R peaks of ECG were detected using the Pan-Tompkin algorithm (Pan and Tompkins, 1985) and were used to segment each SCG signal into cycles.
- Considering scg_*i*_ and scg_*j*_ as the i^*th*^ and j^*th*^ cycles of the SCG signal, respectively, the warping paths, iSCG_*i*_ and iSCG_*j*_, that minimized the total Euclidean distance between scg_*i*_(iSCG_*i*_) and scg_*j*_(iSCG_*j*_), were calculated via the Dynamic Time Warping (DTW) (Müller, 2007).
- M_*corr*_ was estimated as:
  (1) $$\begin{array}{r} M_{corr}\left(i,j\right)=normCorr\left(scg_{i}\left(iSCG_{i}\right),scg_{j}\left(iSCG_{j}\right)\right) \end{array}$$
  where M_*corr*_ is a n by n matrix and n is the number of cycles in the SCG recording. Each cell M_*corr*_(i, j) represented the DTW cross-correlation between scg_*i*_ and scg_*j*_ which is the normalized cross-correlation between scg_*i*_(iSCG_*i*_) and scg_*j*_(iSCG_*j*_).
- The average of each column of M_*corr*_ was estimated and the cycle scg_*max*_ was chosen as the cycle with the maximum average.
- The DTW cross-correlation between all cycles and scg_*max*_ were calculated and all the cycles with a DTW cross-correlation value larger than the maximum average were grouped to form a family. The rest of the cycles were discarded and all further analysis were applied to the cycles in the family.

The families of cycles were formed for the SCG signals recorded during rest and immediately after the exercise test.

### 2.3. Feature Extraction

From each family of cycles, several features categorized as morphological, temporal, spectral, non-linear, and time-frequency features were extracted (Table 1).

**Table 1:** Description of the features extracted from the family of SCG cycles.

**Feature**	**Description**
**Morphological features**	
meanAmp_*mc*−*im*_	The average of amplitude of MC to IM of all SCG cycles within a family
meanAmp_*im*−*ao*_	The average of amplitude of IM to AO of all SCG cycles within a family
Ratio	The ratio of meanAmp_*im*−*ao*_ to meanAmp_*mc*−*im*_
**temporal features**	
meanHR	The average of the length of all SCG cycles within each family
sdHR	The standard deviation of the length of all SCG cycles within each family
Zero_*cross*_	The rate of zero crossing of all SCG cycles within a family
Eng_*tot*_	The value of total energy of all SCG cycles within a family
En_*eng*_	The value of the energy entropy of all SCG cycles within a family
Skewness	The measure of the symmetry of each family distribution (or the lack of it) around the mean, defined as $skewness=\mu_{3}/\sigma^{3/2}$ where μ_3_ and σ are the third central moment and the standard deviation of each family
kurtosis	The measure of the peakedness (or flatness) of each family distribution, relative to the normal distribution, defined as $kurtosis=\mu_{4}/\sigma^{4}-3$ where μ_4_ and σ are the forth central moment and the standard deviation of each family
**Spectral features**	
nBand1	The ratio of the power in the frequency band with the frequencies <10 Hz to the total power
nBand2	The ratio of the power in the frequency band with the frequencies >10 Hz and <20 Hz to the total power
nBand3	The ratio of the power in the frequency band with the frequencies >20 Hz and <30 Hz to the total power
**Non-linear features**	
sampEnt (tau, m, r)	The value of sample entropy of all SCG cycles within a family with the embedding delay of tau = 1, 5, 10, 15, 20, the embedding dimension of m = 3, and the cutoff radius of r = 0.2 × standard deviation of time series
ApEnt (tau, m, r)	The value of approximate entropy of all SCG cycles within a family with the embedding delay of tau = 1, 5, 10, 15, 20, the embedding dimension of m = 3, and the cutoff radius of r = 0.2 × standard deviation of time series
corrDim (tau, m)	The value of correlation dimension of all SCG cycles within a family with tau = 1, 5, 10, 15, 20, and m = 3
**Time-frequency features**	
wavEnt(j)	The wavelet entropy of all SCG cycles within a family at resolution levels of j = 1 .. 4, using “db4” mother wavelet (Rosso et al., 2001)
wavEnt_*tot*_	The total wavelet entropy of all SCG cycles within a family

#### 2.3.1. Morphological Features

**Figure 2:**
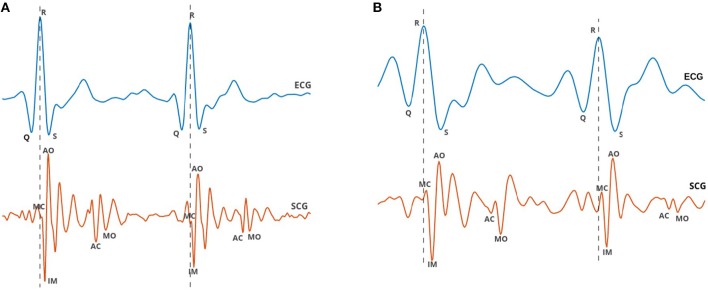
ECG and SCG signals were simultaneously recorded from a 67-year-old male participant with two arteries occluded more than 50%: **(A)** during rest and **(B)** immediately after exercise. Characteristic points of SCG labeled as MC, IM, AO, AC, and MO coincide with mitral valve closure, isovolumic contraction, aortic valve opening, aortic valve closure, and mitral valve opening, respectively.

Using an automated algorithm for delineation of SCG, proposed by Khosrow-Khavar et al. (2016) the fiducial points of each SCG cycle including MC, IM, AO, AC, and MO were located (Figure 2). These fiducial points are suggested to be associated with mitral valve closure (MC), isovolumic contraction (IM), aortic valve opening (AO), aortic valve closure (AC), and mitral valve opening (MO), respectively (Crow et al., 1994). Three features were extracted including the average of amplitude of MC to IM of all SCG cycles within a family, the average of amplitude of IM to AO and the average of the ratio of the amplitude of IM to AO to the amplitude of MC to IM.

#### 2.3.2. Temporal Features

The average and standard deviation of the length of all SCG cycles within each family were estimated. In addition, the rate of zero crossing, total energy, energy entropy, skewness, and kurtosis of each family were estimated.

#### 2.3.3. Spectral Features

The power spectral density (PSD) of a SCG family was estimated using Burg's method through an autoregressive modeling with 1,024 points and an order of 16. The power in each of the following frequency bands was computed by determining the area under the PSD curve bounded by the band of interest: Band1 with frequencies >10 Hz, Band2 with frequencies >10 and <20 Hz, and Band3 with frequencies >20 and <30 Hz. Three features were extracted from PSD including the ratio of the power in Band1 to the total power, the ratio of the power in Band2 to the total power and the ratio of the power in Band3 to the total power.

#### 2.3.4. Non-linear Features

For each family of cycles, the sample entropy, approximate entropy and the correlation dimension were estimated.

#### 2.3.5. Time-Frequency Features

The wavelet decomposition of the family of beats was estimated at resolution levels of j = 1 .. 4, using “db4” mother wavelet. The wavelet entropy at each level j and then the total wavelet entropy were estimated as defined by Rosso et al. (2001).

#### 2.3.6. Combined Features

To form the data set for training the CAD_*exrc*_ classifier, the features extracted from the rest SCG cycles and the exercise SCG cycles were combined. To consider the variation between the SCG signals recorded during the rest and after exercise, the difference between each pair of rest and exercise features were estimated and added to the row of features as follows:

(2) $$\begin{array}{r} diffFeature_{i}=\frac{\left(restFeature_{i}-exrcFeature_{i}\right)}{\left(restFeature_{i}+exrcFeature_{i}\right)/2} \end{array}$$

where restFeatures_*i*_ and exrcFeatures_*i*_ are the i*th* feature from the rest and exercise set of features, respectively. Subsequently, the data set used to train the CAD_*exrc*_ classifier contained the rest features, the exercise features and the combined features.

### 2.4. Statistical Learning

#### 2.4.1. Model Development and Validation

Least absolute shrinkage and selection operator (LASSO) method was employed to select the relevant features and to develop the final binary multivariate logistic regression classifiers, CAD_*rest*_ and CAD_*exrc*_. The LASSO tuning parameter, λ, was adjusted through 5-fold cross-validation (James et al., 2000).

Leave-one-subject-out method was employed to validate the accuracy of the classifiers. In this method, which is the most extreme form of cross-validation, the features of N-1 participants were assigned to the training set and were used to train a CAD classifier. A decision threshold was chosen for the CAD classifier to maximize a weighted classification score defined as [(the number of correct identifications of true positives) + (the number of correct identifications of true negatives)]. The CAD classifier was then applied to the only participant assigned to the test set to predict the probability belonging to the CAD class (predicted risk). Predicted risk above the decision threshold indicated that the individual has classified into CAD class. This procedure repeated N times.

The performance of the CAD classifiers was evaluated in terms of accuracy, sensitivity, specificity, positive predictive value (PPV), and negative predictive value (NPV).

#### 2.4.2. Comparison Between Models

To compare CAD_*rest*_ and CAD_*exrc*_ models, the bootstrap corrected area under the receiver operating characteristic curve (AUC), net reclassification improvement (NRI), and integrated discrimination improvement (IDI) were estimated (Leening et al., 2014).

Bootstrap corrected AUCs were estimated for the CAD_*rest*_ and CAD_*exrc*_ models (Smith et al., 2014). For calculating the bootstrap corrected AUC, 100 bootstrap samples with replacement were generated using the original data set with N participants. The classifiers were developed in bootstrap samples and tested in the original sample. The difference in AUCs, was computed to estimate the optimism and the corrected AUC.

The NRI quantified the net improvement in reclassifying patients with and without CAD using the CAD_*exrc*_ model as compared to the CAD_*rest*_ model.

Rest and exercise discrimination slopes were estimated as the difference in the average predicted risk between participants with and without CAD predicted by CAD_*rest*_ and CAD_*exrc*_ models, respectively. The IDI was quantified as the increased difference between rest and exercise discrimination slopes.

### 2.5. Interpretation of Exercise ECG

ECG recordings were interpreted blindly by an expert. An abnormal exercise ECG was defined as follows: ≥1 mm horizontal or downsloping ST depression or ≥2 mm of upsloping ST depression. If the rest ECG showed ST depression of ≥1 mm, then ≥2 mm of additional horizontal or downsloping ST depression was required to be classified as abnormal.

## 3. Results

### 3.1. Model Development and Validation

The performance of the rest and exercise models obtained through the leave-one-subject-out validation procedure is depicted in Table 2. Regarding accuracy, sensitivity, specificity, positive predictive value (PPV), and negative predictive value (NPV), the CAD_*exrc*_ shows better performance relative to the CAD_*rest*_ model.

**Table 2:** Classification performance for rest SCG (CAD_*rest*_ model), exercise SCG (CAD_*exrc*_ model), and exercise ECG.

**Model**	**Accuracy (95% CI)**	**Sensitivity (95% CI)**	**Specificity (95% CI)**	**PPV (95% CI)**	**NPV (95% CI)**
CAD_*rest*_ (rest model)	74% (66 to 79)	75% (62 to 79)	72% (66 to 86)	84% (76 to 90)	62% (60 to 70)
CAD_*exrc*_ (exercise model)	82% (76 to 87)	84% (76 to 89)	80% (72 to 89)	88% (82 to 94)	70% (63 to 79)
Exercise ECG	65% (58 to 72)	70% (62 to 78)	55% (43 to 69)	75% (66 to 83)	50% (38 to 62)

*CI, PPV, and NPV stand for confidence interval, positive predictive value, and negative predictive value, respectively*.

Regarding the performance of ECG, rest ECG showed ST depression in 7/180 (3.8%) patients. For the 117 patients with ≥50% stenosis in at least 1 coronary artery, the sensitivity of exercise ECG was 70% and for the 63 patients without significant coronary artery stenosis, the specificity for exercise ECG was 55%. Total accuracy of exercise ECG was 65% (Table 2).

Considering the exercise performance 71/180 (39.4%) patients did not achieve 85% of maximal heart rate. Although the sensitivity of exercise ECG decreased in those patients to 60%, the sensitivity of exercise SCG did not change significantly (86%).

### 3.2. Comparison Between Models

**Figure 3:**
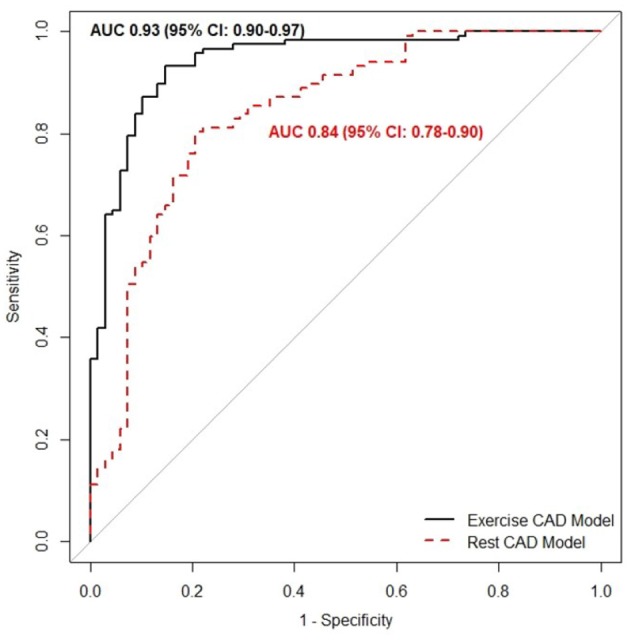
The area under the curve of the receiver operating characteristic of the CAD_*rest*_ (dashed line), which was trained over the features extracted merely from the rest SCG cycles, and the CAD_*exrc*_ model (solid line), which was trained over the features extracted from the rest and immediately after exercise SCG cycles.

The areas under the curve (AUC) of the CAD_*rest*_ and CAD_*exrc*_ models are depicted in Figure 3. The CAD_*exrc*_ model has the higher AUC compared to the CAD_*rest*_ (*p*-value < 0.001). The bootstrap-corrected AUC of the CAD_*rest*_ was 0.77 (95% CI 0.75 to 0.89); for the CAD_*exrc*_ it was 0.88 (95% CI 0.86-0.90).

The CAD_*exrc*_ classifier exhibited a 10% net improvement in classification of patients with CAD and a 6% net improvement in patients without CAD at a decision threshold of 0.60. In the other word, a SCG stress test would increase the number of true positives and also decrease the number of false negatives. However, the increase in the number of true positives was more significant compared to the decrease in the number of false negatives.

**Figure 4:**
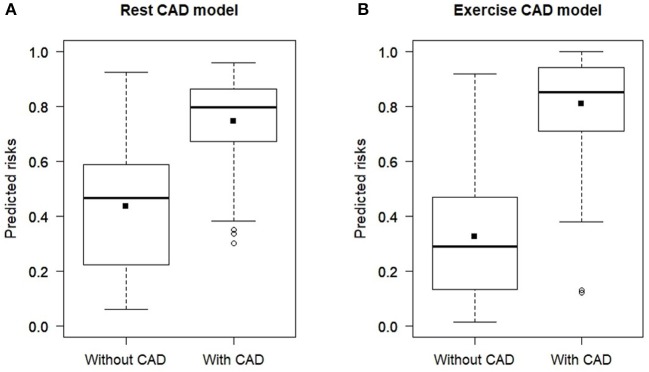
Box plots of predicted risk of individuals with and without CAD estimated by **(A)** CAD_*rest*_ and **(B)** CAD_*exrc*_. The discrimination slope, which is the difference between the mean predicted risk for individuals with and without CAD, was estimated as 0.3 using the CAD_*rest*_ model and 0.5 using the CAD_*exrc*_ model.

Figure 4 shows the predicted risk for individuals with and without CAD estimated using the CAD_*rest*_ and CAD_*exrc*_ models. The discrimination slope was estimated as 0.32 using the CAD_*rest*_ model and 0.47 using the CAD_*exrc*_ model. The difference between discrimination slopes of these two models which is equivalent to integrated discrimination improvement was 0.15 (95% CI: 0.10 to 0.23, *p*-value < 0.0001).

## 4. Discussion

We have developed and validated a prediction model, CAD_*exrc*_, that uses the mechanical activity of the heart, recorded during rest and immediately after exercise, to identify patients with more than 50% occlusion in at least one coronary artery. The features used to develop the model were derived from families of similar cardiac cycles. This model delivered a bootstrap-corrected AUC of 0.88 (95% CI: 0.86 to 0.90) and provided a significant improvement (*p*-value < 0.001) in classification performance relative to the model using the features of the rest SCG cycles, with a bootstrap corrected AUC of 0.77 (95% CI: 0.75 to 0.89).

It was anticipated that in identifying the patients with CAD, the CAD_*exrc*_ model would reveal better performance compared to the CAD_*rest*_ model. During exercise, the oxygen demand of the heart muscle increases. To fulfill this higher demand, the coronary arteries dilate so more oxygenated blood can be transported to the heart muscle. However, if coronary arteries are affected by arteriosclerosis, their dilation capacity is decreased. This may lead to insufficient oxygen supply to the heart, causing myocardial ischemia. Consequently, contractility and motion of the myocardium are decreased. Other reason accounted for difficulties of detecting CAD using rest SCG may be due to the dilation of coronary arterioles. The coronary arterioles arising from the stenosed artery would normally dilate in response to a decrease in blood flow. It would explain why coronary blood flow at rest is not reduced in patients even with 70% stenosis in a single coronary artery (Uren et al., 1994). However, it only applies to the branched vessels and is only relevant if the basal myocardial oxygen demand remained constant, for example during the rest.

Although the findings of the current study showed that the performance of rest SCG was not as high as the exercise SCG in identifying patients with CAD, however, the rest SCG reached better performance compared to the exercise ECG (Table 2). The sensitivity and specificity of the rest SCG were 75 and 72%, respectively, which were higher compared to those calculated for the exercise ECG (70 and 55%, respectively, for sensitivity and specificity). These findings were supported by the results of other studies as well. Several studies showed that the sensitivity of the exercise ECG ranged between 68 and 75% (Al-Shehri et al., 2011; McLellan and Prior, 2012) and the specificity ranged from 70 to 77% (McLellan and Prior, 2012). Besides, there is a considerable drawback that an exercise ECG test can only be performed by a trained physician. In contrast to the exercise ECG, a rest SCG can be recorded by individuals without the medical background.

In a study conducted by Salerno et al. an exercise SCG was suggested for detecting CAD and its accuracy was evaluated alone and in conjunction with an exercise ECG (Salerno et al., 1992). The authors analyzed the changes in waveform morphology and waveform amplitude of the SCG that occurred between the recordings prior to and immediately after exercise. The proposed method was validated on the same set of patients initially used to develop the method and not in a separate test data set. The results showed a sensitivity and specificity of 80 and 69%, respectively, for detection of ≥50% coronary artery stenosis. In the current study, our suggested method was trained and validated in the separate data sets and still achieved the better performance, with higher sensitivity and specificity matrices relative to the performance reported by Salerno et al. (1992). In addition, Salerno et al. did not report the performance of the rest SCG. In the same study, sensitivity and specificity were reported as 67 and 51%, respectively, for the exercise ECG. Comparing these results shows that the exercise SCG provided better performance in identifying patients with CAD compared to the exercise ECG. Even the rest SCG contributed to a more accurate implementation in classifying patients with suspected CAD, conducted in the same data set.

An evidence-based analysis of more than 120 publications was recently conducted to determine the accuracy of stress echocardiography with regard to CAD. Overall pooled sensitivity of 80% (95% CI: 0.77–0.82) and specificity of 84% (95% CI: 0.82–0.87) were reported using coronary angiography as the reference standard (Medical Advisory Secretariat, 2010). In our study, exercise SCG showed similar results in terms of sensitivity and specificity compared to those reported for the stress echocardiography. In our opinion, performing an exercise SCG is more convenient than performing a stress echocardiography. Furthermore, analysis of SCG recordings is much easier than interpreting echocardiographic images.

Performance of the exercise SCG is also comparable with the performance of coronary computed tomography angiography (CCTA). Sensitivity and specificity of CCTA are stated to be between 85 and 90% and 64 and 90%, respectively. However, CCTA has a very high negative predictive value, especially in low to intermediate risk subjects (Al-Shehri et al., 2011). Furthermore, CCTA is only available in specialized centers and it is by far more expensive compared to SCG examination.

In the current study, instead of analyzing all cycles of each SCG recording, we selected a group of similar cycles, the so-called family of cycles. Family selection reduces the cycle to cycle variation of SCG morphology by finding the cycles with a high level of similarities. The variability in the morphology of SCG cycles is mainly due to the effect of breathing which is more severe after exercise. As a result, family selection step was essential to select the main representative morphology for each participant. In fact, our study showed a significant increase in the overall accuracy of the algorithms with the family selection procedure.

The findings of the current study may suggest two possible scenarios for the use of SCG in the detection of CAD: (1) SCG obtained with exercise ECG in the clinical setting and, (2) resting SCG and ECG recorded at home in a portable stand-alone solution. Similar to an exercise ECG, the exercise SCG would be restricted to medical facilities under the supervision of a trained physician (e.g., a cardiologist) due to the risk of a stress induced cardiac event. However, as reported by Salerno et al. (1992) and supported by this study, the combination of exercise SCG with exercise ECG would increase the sensitivity of detecting CAD. Accordingly, if this technology is used in the clinical setting, the combination of exercise SCG would increase the sensitivity of the ECG stress test. Furthermore, the results of the current study showed that the performance of the rest SCG was comparable to the performance of the exercise ECG. In addition, our results showed that the sensitivity of exercise SCG did not change significantly in the patients who did not achieve 85% of maximal heart rate while the sensitivity of exercise ECG dropped dramatically from 70 to 60% in those patients. It would suggest that for detecting CAD the exercise SCG test may not need to reach the maximal effort. In other words, it could be possible to develop a portable at-home screening tool for coronary artery disease based on the SCG recorded during the rest or after a very moderate effort, such as a fast walk approved by a physician.

### Future Work

In our future studies, we aim to address the following limitations of the current study: (a) analysis of other accelerometer axis as well as rotational gyroscope. In the current study, we only analyzed the z-axis of the accelerometer signal in the dorsoventral direction. However, the movement of the chest due to cardiac vibration is not limited to this direction. It may also manifest itself in the other two accelerometer axes and also in rotational movements, which can be picked up by gyroscopes (Jafari Tadi et al., 2017). We intend to record these additional signals in the future study. With this technique, we hope to increase sensitivity, specificity, and accuracy of CAD identification; (b) analysis of stenosis with different degree of occlusion. In the current study, we investigated the possibility of identifying patients with stenosis ≥50% in at least one coronary artery. In a future study, we will investigate the possibility of early detection of the individuals with coronary artery stenosis of a 25% occlusion rate. Also, we will investigate the potential of SCG in localizing the coronary occlusion; (c) exploring the rest SCG as a stand-alone solution for detection of CAD. Since conducting an exercise SCG is not feasible without clinical control, in the future study we will investigate the ability of resting SCG to detect CAD as a stand-alone solution; and, (d) exploring different exercise levels required for detection of CAD. We will explore the minimum intensity of the exercise load needed to provide a performance comparable with exercise SCG at the maximum load conducted in a clinical setting. This would provide the data needed for the development of a portable at-home CAD screening solution using SCG.

In conclusion, we found that rest SCG and exercise SCG are able to identify patients with coronary artery stenosis ≥50%. The performance of SCG is better compared to the exercise ECG and is more or less identical with stress-echocardiography and CCTA. SCG is faster and less expensive and can even be carried out by individuals without any medical background. However, further studies are necessary in order to prove that SCG under resting conditions is a sufficient stand-alone solution for identifying coronary artery disease.

## Data Availability Statement

The datasets generated for this study will not be made publicly available. Currently, this data set is in the possession of Heart Force Medical Inc. However, the authors have the intention to make the data set publicly available in the near future. The features extracted from the signals are available by request.

## Ethics Statement

The studies involving human participants were reviewed and approved by the human subject research committee of Abbott North-western Hospital, Minneapolis, MN, on March 11, 1988. The patients/participants provided their written informed consent to participate in this study.

## Author Contributions

PD processed the data, designed and developed the models, analyzed the results, prepared the figures, and drafted the manuscript. EB provided his medical expertise in designing the models and statistical processing, analyzed the results and also revised the manuscript critically for content. KT, VZ, and AB analyzed the results and revised the manuscript critically for content. FK-K contributed to the design and development of the models and revised the paper critically for content.

### Conflict of Interest

PD and VZ are employed by Heart Force Medical Inc., Vancouver, Canada. KT and EB are on the Board of Directors at Heart Force Medical, Inc. Vancouver, Canada. FK-K is the CTO of Heart Force Medical Inc., Vancouver, Canada. The remaining author declares that the research was conducted in the absence of any commercial or financial relationships that could be construed as a potential conflict of interest.
